# Post-Processing Treatment Impact on Mechanical Properties of SLM Deposited Ti-6Al-4 V Porous Structure for Biomedical Application

**DOI:** 10.3390/ma13225167

**Published:** 2020-11-16

**Authors:** Eren Pehlivan, Jan Džugan, Jaroslav Fojt, Radek Sedláček, Sylwia Rzepa, Matej Daniel

**Affiliations:** 1 Department of Mechanics, Biomechanics and Mechatronics, Faculty of Mechanical Engineering, Czech Technical University in Prague, Technická 1902/4, 16000 Prague, Czech Republic; eren.pehlivan@fs.cvut.cz (E.P.); Radek.Sedlacek@fs.cvut.cz (R.S); 2 Mechanical Testing and Thermophysical Measurement Department, COMTES FHT a.s., Průmyslová 995, 33441 Dobrany, Czech Republic; jan.dzugan@comtesfht.cz (J.D.); sylwia.rzepa@comtesfht.cz (S.R.); 3 Department of Metals and Corrosion Engineering, University of Chemistry and Technology, Technická 5, 16628 Prague, Czech Republic; Jaroslav.Fojt@vscht.cz

**Keywords:** selective laser melting, titanium alloy, additive manufacturing (AM), surface treatment, hot isostatic pressing (HIP)

## Abstract

Additive manufacturing technologies allow producing a regular three-dimensional mesh of interconnected struts that form an open-cell porous structure. Regular porous structures have been used in the orthopedic industry due to outstanding bone anchoring. The aim of the study was to determine how the postprocessing influences the mechanical properties of porous structures made of titanium alloy CL 41TI ELI. The effect of hot isostatic pressing (HIP) as a method of increasing microstructural integrity was investigated here. The influence of surface etching (SE) technique, which was applied to the porous structure for cleaning unmelted titanium powder particles on the surface of connectors from the inner surfaces of a porous structure, was examined in this study. Mechanical properties were investigated by means of compression tests. The results point out that HIP has a minor effect on the mechanical behavior of considered porous structures. The SE is an effective method to clean the surface of a porous structure, which is very important in the case of biomedical applications when loose powder can cause serious health problems. Another effect of the SE is also the strut thickness reduction. Reducing strut thickness of a porous structure with the surface etching decreases its stiffness to the same extent as predicted by the relative density theoretical model but did not result in structural damage.

## 1. Introduction

Additive Manufacturing (AM) is an innovative, rapidly developing technology utilized in the biomedical industry. The AM provides a custom shape freedom with lightweight structure optimization for replacing injured or diseased joints [1,2,3,4]. Additively manufactured joint and bone replacements are mostly made of biocompatible titanium and its alloys. However, solid titanium alloys are characterized by significantly higher stiffness than human bones. This mismatch in mechanical properties could limit bone ingrowth, speed up bone resorption and cause loosening of the orthopedic implant as a result of stress shielding [5,6,7]. The phenomenon is a reaction of bone to changing load conditions by its remodeling. The small porous and open-cell structures are arranged as repeating and connected unit cells and can be created by AM. This kind of architecture tends to reduce a stress gradient between the bone and the implant, and thus, promote a bone in-growth and improve osteointegration [8,9,10,11,12]. The mechanical properties of cellular structures can be further altered by an adjustment of open-cell architecture features, such as a strut thickness (relative density) and a material choice [13,14,15,16,17].

Three-dimensional porous structures can be prepared by using titanium powder by selective laser melting (SLM) belonging to powder bed fusion (PBF) technology [18,19,20,21,22,23]. In this system, the deposition process is conducted under a protective atmosphere of inert gas. Implants can be fabricated by melting a powder layer-by-layer using a laser beam as an energy source. After the deposition of a single material layer, a powder bed goes downwards, and the next powder layer is spread by a roller or re-coater. The process is continued until a whole structure is formed. The deposited bulk material could contain internal pores and defects as a result of gas entrapment in the powder particles or SLM deposition parameters selection [24,25]. These internal defects influence the static and fatigue strength, considerably acting as stress concentrators [13]. In other studies, it was demonstrated that hot isostatic pressing (HIP) could be an effective way of porosity reduction in AM-ed metals and their alloys [26,27,28]. Ahlfors et al. investigated the influence of HIP on the AM-ed titanium alloy [28]. The authors concluded that the fatigue performance and ductility of deposited parts could be significantly enhanced as a result of HIP treatment. While the influence of the HIP process is broadly documented for bulk structures, its potential advantage for open-cell architectures has not been extensively described yet.

The SLM method, as a layered fabrication process, produces not only internal defects but also a very rough surface. According to the research of Harun et al., a rough and highly porous structure potentially promotes the bone in-growth and on-growth [29]. Nonetheless, during the deposition process, some powder particles remain not fully melted and loosely attached to the implant surface. These particles remarkably increase the risk of contamination after surgery. In other studies, it was proposed that the released titanium particles can have adverse long-term metabolic, oncogenic and immunologic effects [30,31,32]. Mombelli et al. concluded that the reaction of human tissues to the adjacent titanium implant is very complex, where the presence of titanium particles, the process of tribo-corrosion and the development of inflammation influence each other [30]. According to the study of Heringa et al., the accumulation of titanium in a human body can lead to liver damage [33]. The research of Woodman et al. also demonstrates an effect of ion release from titanium-based implants as damaging to the vital human organs, namely liver and kidney [34]. It was suggested that the separation of any unmelted powder particle from porous structure could lead to critical liver failure [32]. Thus, a thorough surface cleanup is highly recommended for any product used for commercial orthopedic purposes. The removal of loosely connected powder particles can be conducted by means of machining and polishing (mechanical or chemical).

Due to the increased risk of serious health problems of a patient, the process of implant cleaning is considered essential for further application. It was proposed that a chemical or electrochemical process could be used to remove surface powder [35]. Such a process is not specific to powder and also affects the bulk material, namely strut dimensions and porosity. Therefore, a chemical treatment of porous structure is a stochastic process that depends on the local surface quality and could considerably compromise the mechanical properties of porous samples. However, the surface etching slightly affects surface chemistry and thus corrosion behavior. The corrosion rate is higher than in the case of a non-etched surface. Nevertheless, cytocompatibility remains at the same level as for untreated surfaces [36].

**Figure 1:**
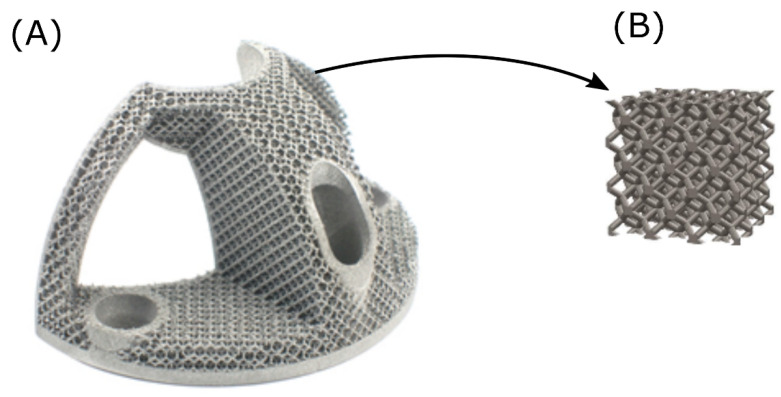
(**A**) Acetabulum metal augments implant with rhombic dodecahedron porous structure surface and (**B**) rhombic dodecahedron cubical element [37].

In this study, a porous design of cubical testing specimens was derived from the commercially available acetabulum augments implant, as presented in Figure 1. The surface was cleaned by chemical etching for as-built, and HIP treated specimens. The aim of this study was to determine if and to what extend (a) the HIP treatment and (b) the chemical etching affects the mechanical properties in porous specimens.

## 2. Materials and Methods

**Figure 2:**
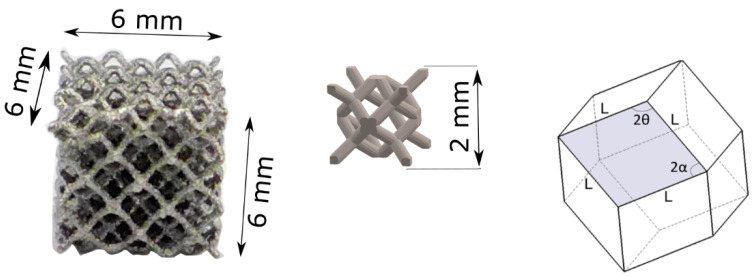
Compression specimen dimensions (**left**), a single unit cell dimensions (**middle**) and rhombic dodecahedron unit-cell (**right**).

The specimens were fabricated using Concept Laser titanium alloy grade 23 (CL 41TI ELI, Concept Laser GmbH, Lichtenfels, Germany) powder [38]. In order to design the computer models of cubical specimens, a computer-aided design package SolidWorks (Version 27, Dassault Systemes SolidWorks Corp., Waltham, MA, USA) and Materialise/Magics (Magics, version 23, Leuven, Belgium) software were employed. The dimensions of a cubical specimen were set up as 6 mm with a single rhombic dodecahedron open unit-cell of 2 mm, as can be seen in Figure 2. An individual rhombic dodecahedron unit cell is composed of 12 identical rhombic faces with 24 edges and 14 vertices [17]. The length of the strut (l) was 0.87 mm. The constant angles between connectors were designed as 2α = 70.53° and 2θ = 109.47°. The strut was characterized by a thickness of 0.3 mm that yielded a relative density of 20%. The connector struts were designed as circular.

The deposition process was conducted using a M2 fusing machine (Concept Laser GmbH, Lichtenfels, Germany), adopting the SLM method. The SLM process was performed according to the manufacturer’s recommendation. The SLM deposition parameters are summarized in Table 1. Prior to the deposition process, a building chamber was not preheated. Concept Laser’s ‘island’ scanning strategy was applied [39].

The HIP treatment was applied in order to reduce the internal defects, increase structural integrity and potentially improve the mechanical properties of the deposited structures. The HIP was carried out by Bodycote Bourgogne (Bodycote HIP Ltd., Magny-Cours, France). The process was conducted under a protective argon gas atmosphere of 1020 bars. The specimens were heated up to 900 °C in 5 h and held at 900 °C for 4 h in steady isostatic pressure. Subsequently, the specimens were cooled down to room temperature in 5 h. The specimens were divided into 6 batches according to the applied posttreatment method, where each batch contained 4 specimens for statistical analysis. The specimen designation system, the number of specimens and the corresponding postprocessing method are summarized in Table 2.

The surface etching was aimed to decrease the surface roughness and to remove partly unmelted surface powders. The surface treatment started with 5 min degreasing in ethanol in an ultrasonic bath. Then, the specimens were etched for a defined time (3 min or 6 min) in a solution of 20 mL HF, 200 mL HNO_3_ and 780 mL demineralized water in the ultrasonic bath. The specimens were etched in batches of six pieces so that the etching bath volume was high enough to guarantee the success of the process. The specimens were subsequently washed with demineralized water (in the ultrasonic bath) and finally dried by air steam.

The etching conditions were selected based on the previous study of authors. Six minutes is the minimal time to achieve a surface with no partially melted particles [36]. The measurements of the cross-sectional area of the cubical specimens were performed by means of a digital micrometer (Mitutoyo, Mitutoyo Corporation, Tokyo, Japan) for all specimens.

In order to determine a geometrical accuracy and thus, identify the effect of applied postprocessing, a connector strut thickness and cross-sectional area were measured using a scanning electron microscope (SEM) Tescan VEGA-3 LMU (Tescan, Brno, Czech Republic) with ImageJ software (1.52i, NIH, MD, USA) [40]. The SEM is based on a secondary electron (SE) signal detection and operates at 20 kV. The strut thickness was measured on two specimens per batch. Forty-five measurements per specimen were performed that corresponds to approximately two records per each strut.

**Figure 3:**
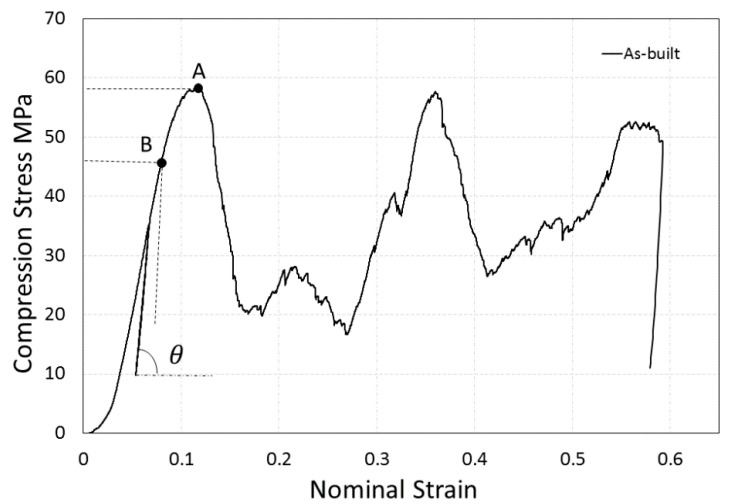
Elastic gradient calculation in accordance with the standard ISO 13,314. The compression stress–strain diagram with a marked elastic gradient tangent of ‘’θ’’. A 0.2% offset compressive proof stress is designated as “B” and maximum first stress is designated as “A” [37].

The mechanical compression tests were carried out with MTS 858 Mini Bionix testing machine (MTS, Eden Prairie, MN, USA) with a load cell of capacity 5 kN. The loading speed of a crosshead was set up as a constant 0.1 mm/min. The elastic gradient was determined in accordance with the standard ISO 13314 and calculated as the slope of the stress–strain curves between 30% and 70% of the plateau strength. The compressive proof stress and maximum first strength values were determined from the diagram using a 0.2% offset method, as is shown in Figure 3.

Theoretical relative density, elastic modulus and yield strength were calculated based on A. A. Zadpoor et al., R. Hedayati et al. and S. M. Ahmadi et al. studies [41,42,43].

The relative density formula is [42]:

(1) $$\rho =\frac{3\sqrt{3}}{2}\pi {\left(\frac{r}{l}\right)}^{2}-\frac{27\sqrt{2}}{4}\pi {\left(\frac{r}{l}\right)}^{3}$$

The analytical elastic modulus formula is [43]:

(2) $$\frac{E_{1}}{E_{s}}=\frac{E_{2}}{E_{s}}=\frac{27\frac{sin\theta }{sin2\theta }}{\frac{3l^{4}}{\pi r^{4}}+\frac{18l^{2}}{\pi r^{2}}}$$

The analytical yield stress modulus formula is [42]:

(3) $$\frac{\sigma y_{1}}{\sigma y_{s}}=\frac{\sigma y_{2}}{\sigma y_{s}}=\frac{\sqrt[{4}]{3}}{4}\rho^{\frac{3}{2}}$$

## 3. Results

**Figure 4:**
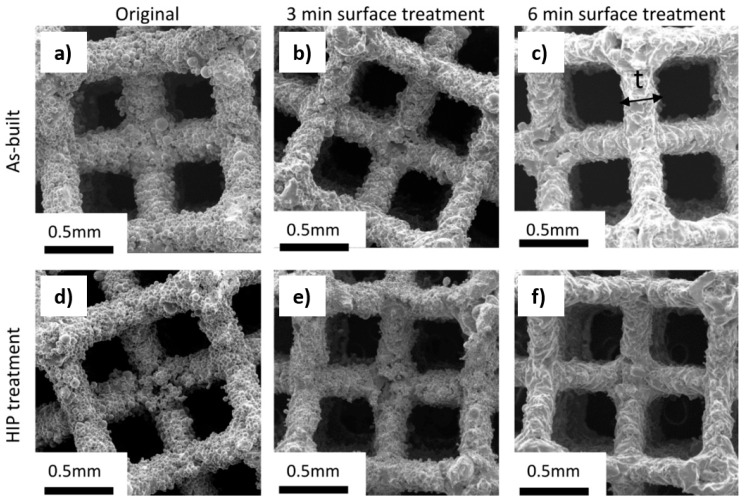
Secondary electron micrographs of the representative specimens selected from each tested batch. Comparison of the postprocessing effect on a specimen surface (batch/strut thickness in (×0.001) mm): (**a**) as-built specimen with original surface (B/322 ± 21); (**b**) as-built specimen after 3 min etching (B3/277 ± 21); (**c**) as-built specimen after 6 min etching (B6/243 ± 21); (**d**) HIP-ed specimen with original surface (H/310 ± 23); (**e**) HIP-ed specimen after 3 min etching (H3/287 ± 21); (**f**) HIP-ed specimen after 6 min etching (H6/234 ± 18) [37].

**Figure 5:**
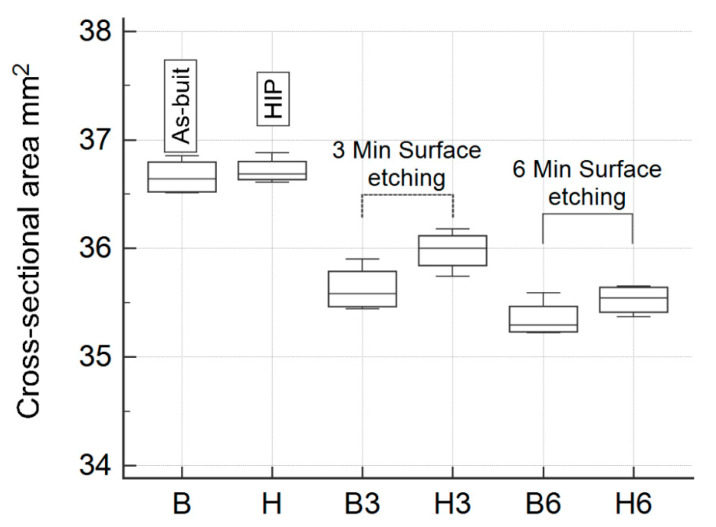
Comparison of the cross-sectional area of porous specimens subjected to various postprocessing methods [37].

Figure 4 presents the surface etching effect on the specimens in as-built and HIP-treated states. The surface etching caused the removal of partly melted powder particles from the struts and the creation of a smoother surface. Removing of a covering layer of the surface resulted in a reduction of the strut thickness up to 14%, as presented in Table 2. The struts subjected to the etching achieved a clear beam form and homogenous shape. The HIP treatment resulted in a reduction of the strut diameter slightly. The decrease in the cross-sectional area could be observed not only at the strut level but also at the porous specimen total cross-sectional area, as summarized in Figure 5. A comparison of the strut thicknesses with corresponding mechanical characterictics obtained experimentally and using analytic model for the specimens in as-built, HIP-ed and etched states is presented in Table 3.

**Figure 6:**
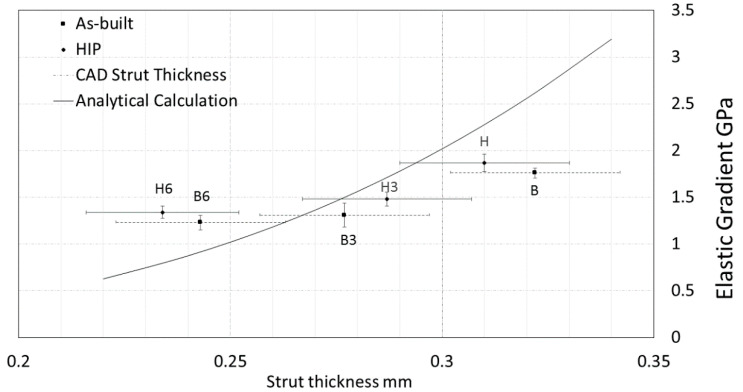
The effect of posttreatment on strut thickness and elastic gradient of the porous structure. The analytical model prediction is based on the change of strut thickness [37].

The elastic gradient was calculated in accordance with the ISO 13,314 guidelines by elastic loading and unloading [44]. The observed decrease in the elastic gradient trend may be caused by the reduced strut thickness, as it was predicted by the analytical model. The specimens after HIP treatment (H) achieved a 5.88% higher elastic modulus value than as-built specimens (B). The lowest elastic gradient was recorded for the specimens after 6 min surface treatment (B6) (1.23 ± 0.08 GPa), as presented in Figure 6.

**Figure 7:**
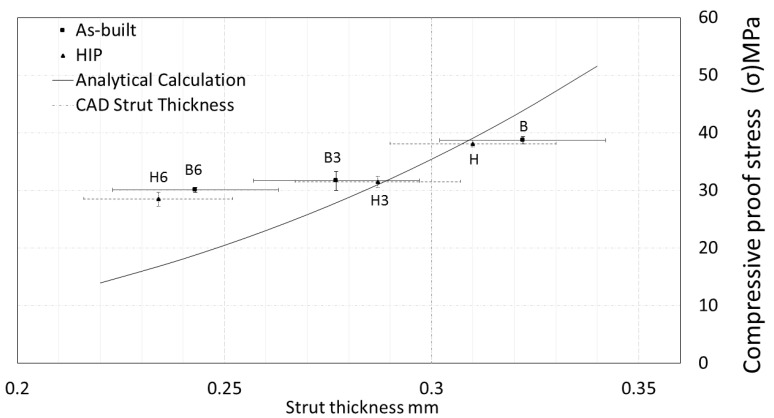
The effect of posttreatment on strut thickness and compressive proof stress of the porous structure. The analytical model prediction is based on the change of strut thickness [37].

The strut thinning that was a result of the surface etching led to a decrease of compressive proof stress as expected by analytic calculation. The compressive proof stress of porous specimens was not significantly affected by HIP. The specimens after both HIP and 6 min surface treatment (H6) were distinguished by the lowest level of compressive proof stress that reached 28.49 ± 1.24 MPa, as can be seen in Figure 7.

## 4. Discussion

Strut thickness and cross-sectional area influence the mechanical performance of open-cell porous structures [45]. In the present study, the surface etching led to a decrease of the strut thickness (Table 2), and thus, the mechanical response of the porous structure has changed accordingly, as presented in Figure 6 and Figure 7. The observed trend is in agreement with theoretical predictions. The predicted values are within the same range as the experimentally measured ones for as-built, HIP and 3 min etched specimens. However, the mechanical parameters for the specimens after 6 min etching are slightly higher than the values predicted on the basis of the strut thickness. It indicates that other mechanisms than a simple reduction of the strut thickness should be considered. For example, it could be assumed that the joints of the unit cell are not affected by etching proportionally (Figure 4) that is not included considerably by the analytic calculation.

The etching may be promoted on the surface defects of individual struts that could potentially lead to local weakening. An inconsistent structure can provide a nonuniform deformation while compressive loading, which suggests that the equivalent connector struts deal with the different loading. Such local structural differences would result in various mechanical response observed between the individual specimens within the same group exhibited as a large variance within the group. Within the present study, such behavior was not confirmed. Despite rough structure in as-built specimens (Figure 3), the recorded values exhibited relatively low data scatter. The surface roughness contributes to the deviation of strut diameter values; nonetheless, the variations between the mechanical response of the specimens increased only slightly (Figure 6 and Figure 7).

The specimen post-deposition treatment can affect the effectiveness of surface etching. The specimens in the as-built state, subjected to 3 min etching (B3), were characterized by a reduced relative density by 7.27% in comparison to the specimens in an initial state (B). In the case of the HIP-ed specimens (H and H3), the etching led to relative density reduction by 3.70%, as shown in Table 2. In addition, the total change in relative density after 6 min etching was 12.03% and 11.14% for the specimens in as-built (B6) and post-treated (H6) states, respectively. A similar trend can be observed in overall porous specimen dimensions, as illustrated in Figure 5.

According to Song et al. [46] and Oh et al. [47], a reduction in internal defects should considerably increase the elastic modulus of a solid structure. Furthermore, HIP treatment also decreases internal defect size and increase the elastic gradient. Although a similar behavior was recorded for porous structure in the present study, the observed effect was minor. The highest value of the elastic gradient was noticed for the unetched batch subjected to the HIP treatment (H), and it was only 5% higher than for the porous specimens in the as-built state (B). On the other hand, the lowest elastic gradient values were recorded for the as-built 6 min and 3 min etched batches (B6 and B3), reaching 1.23 and 1.31 GPa, respectively.

In this study, the etched porous structures were assessed to be more compliant for medical applications than those subjected only to the HIP treatment. Obtaining similar values of mechanical characteristics for a human bone and deposited porous structures is desirable for joint replacements and result in the reduction of stress shielding, decrease the risk of implant loosening as well as an extension of the implant lifetime [48]. Surface etching also provides an excellently clean product (Figure 4), free of unmelted powder surface particles that could potentially lead to the damage of vital human organs. Preventing the release of titanium particles from the implant is essential for any future biomedical application.

## 5. Conclusions

In the present study, the effect of HIP treatment and surface etching on the mechanical properties of porous specimens was investigated. It was shown that HIP processing is not considered effective for porous structures. However, surface etching appears to be an appropriate method as a post-deposition treatment of the porous structures for biomedical implants. By erosion of loosely attached particles from the surface, it prevents contamination of the human body by titanium particles. Furthermore, it lowers the elastic gradient and provides consistent mechanical properties. The material removed by etching slightly decreases the strength of the whole porous structure due to load-bearing cross-section reduction.

## 6. Future Works

Reflecting on an application of open-cell structures in biomedicine, many other factors must be taken into consideration and investigated in detail. The presented examination was performed with a static compression test in ambient conditions. However, the porous structures being implanted into the human body are supposed to deal with dynamic loading conditions in blood-like material and the environment of body fluids. These fluids and cells penetrate the porous structure that can induce a corrosion process of the implant [36,49]. This effect can lead to disruption of the physiological ion movement in human nerve cells. In addition, in vivo environment can lead to an increase of the open-cell structure density and strength as a result of the interpenetrating phase composites effect [50].

## Figures and Tables

**Table 1:** Summary of applied selective laser melting (SLM) deposition parameters.

Deposition Parameters	
Laser beam power	200 W
Scan speed	7 mm/s
Layer thickness	20 µm
Offset distance	75 µm

**Table 2:** Experiment plan-specimen designation system, corresponding group definition and number of specimens per conditions.

Group Designation	Postprocessing Parameters	Number of Specimens
B	No heat (as-built)	4
H	HIP treatment	4
B3	Surface treatment 3 min	4
B6	Surface treatment 6 min	4
H3	HIP treatment and surface treatment 3 min	4
H6	HIP treatment and surface treatment 6 min	4

**Table 3:** Comparison of strut thickness and mechanical parameters measured and predicted by the analytic model for the specimen batches subjected to various postprocessing methods.

Gr.	Strut Thickness (×0.001) mm	Relative Density%	Compressive Proof Stress Theoretical MPa (σ)	Compressive Proof Stress Experiment MPa (σ)	Elastic Gradient Theoretical GPa (E)	Elastic Gradient Experiment GPa (E)
B	322 ± 21	27.94	43.86	38.70 ± 0.63	2.62	1.76 ± 0.05
B3	277 ± 21	20.67	27.92	31.66 ± 1.66	1.50	1.31 ± 0.13
B6	243 ± 21	15.91	18.85	30.11 ± 0.43	0.92	1.23 ± 0.08
H	310 ± 23	25.89	39.14	38.13 ± 0.55	2.28	1.87 ± 0.05
H3	287 ± 21	22.19	31.05	31.50 ± 0.94	1.71	1.48 ± 0.11
H6	234 ± 18	14.75	16.83	28.49 ± 1.24	0.79	1.34 ± 0.06
